# Breaking through the strength-ductility trade-off dilemma in an Al-Si-based casting alloy

**DOI:** 10.1038/srep30874

**Published:** 2016-08-09

**Authors:** B. Dang, X. Zhang, Y. Z. Chen, C. X. Chen, H. T. Wang, F. Liu

**Affiliations:** 1 State Key Laboratory of Solidification Processing, Northwestern Polytechnical University, 710072 Xi’an, P.R. China; 2 Institute of Applied Mechanics, Zhejiang University, 310027 Hangzhou, P.R. China

## Abstract

Al-Si-based casting alloys have a great potential in various industrial applications. Common strengthening strategies on these alloys are accompanied inevitably by sacrifice of ductility, known as strength-ductility trade-off dilemma. Here, we report a simple route by combining rapid solidification (RS) with a post-solidification heat treatment (PHT), i.e. a RS + PHT route, to break through this dilemma using a commercial Al-Si-based casting alloy (A356 alloy) as an example. It is shown that yield strength and elongation to failure of the RS + PHT processed alloy are elevated simultaneously by increasing the cooling rate upon RS, which are not influenced by subsequent T6 heat treatment. Breaking through the dilemma is attributed to the hierarchical microstructure formed by the RS + PHT route, i.e. highly dispersed nanoscale Si particles in Al dendrites and nanoscale Al particles decorated in eutectic Si. Simplicity of the RS + PHT route makes it being suitable for industrial scaling production. The strategy of engineering microstructures offers a general pathway in tailoring mechanical properties of other Al-Si-based alloys. Moreover, the remarkably enhanced ductility of A356 alloy not only permits strengthening further the material by work hardening but also enables possibly conventional solid-state forming of the material, thus extending the applications of such an alloy.

Al-Si-based casting alloys have been widely used for complex-shape components in aerospace and automobile industries because of their excellent castability, low density, corrosion resistant, high strength to weight ratio, and low coefficient of thermal expansion^1^. Under normal casting conditions, the microstructures of Al-Si-based casting alloys consist mainly of soft Al dendrites and brittle eutectic (Al + Si) phase formed in the inter-dendritic regions. The addition of Si is greatly helpful for improving the castability^2^, but the formation of eutectic (Al + Si) phase brings forth detrimental effects on mechanical properties, of the Al-Si-based casting alloys. On the one hand, the coarse Si phase within the eutectic (Al + Si) phase is brittle, making them being suspicious to cracking subjected to stress accumulation at Al/Si interface. On the other hand, the as-solidified Al dendrites contain few dislocation barriers; internal stress produced upon plastic deformation accumulates easily at the Al/Si interface. Once the accumulative internal stress exceeds a critical stress, the cracking of Si will occur and subsequently lead to the fracture of the alloys^3^. Therefore, the Al-Si based casting alloys normally suffer from low strength and low ductility. Various strategies, such as foreign particle reinforcement^4^,^5^,^6^, grain refinement^7^,^8^,^9^,^10^, micro-alloying^11^,^12^ and precipitation hardening^13^, have been employed to improve the mechanical properties of these alloys. However, as shown in Fig. 1 using a commercial Al-Si-based A356 casting alloy as an example, the increase in strength is inevitably sacrificed by the reduction in ductility, which is known as the strength-ductility trade-off dilemma in structural metals^14^,^15^. Although grain refinement realized by rapid solidification^7^,^8^ and addition of grain refiner^9^,^10^, especially rapid solidification, is expected to cause a concomitant increase of strength and ductility; its effect is considered to be limited as the data reported in the literature are still within the shaded area showing the strength-ductility trade-off.

Using a commercial Al-Si-based casting alloy (A356 with a nominal composition of Al-7.0Si-0.4Mg-0.1Fe in wt.%) as an example, we herein reported a simple route by combining rapid solidification (RS) with a post-solidification heat treatment (PHT, i.e. heating the as-solidified alloys at a moderate temperature for a certain time) to break through the strength-ductility trade-off dilemma. It is shown that the application of RS + PHT route does realize the simultaneous increases of strength and ductility of the A356 alloy by increasing the cooling rate upon RS (as shown in Fig. 1).The best combination of yield strength (YS, 0.2% proof stress) and elongation to failure (ETF) achieved by combining the RS + PHT route with the commonly used T6 heat treatment (solid-solution treatment + artificial aging) stands beyond the region showing strength-ductility trade-off behavior (Fig. 1), disobeying the general trend of strength-ductility trade-off of the A356 alloy. This can be ascribed to the hierarchical microstructure formed by the RS + PHT route, i.e. highly dispersed nanoscale Si particles in the interior of Al dendrites and nanoscale Al particles decorated in eutectic Si. The former enhances the work hardening of Al dendrites, while the latter leads to the ductilization of eutectic Si phase. On the one hand, the simplicity of the developed RS + PHT route enables the possible applications for industrial scaling production, and on the other hand, the strategy of engineering microstructures offers a general pathway in tailoring mechanical properties of other Al-Si-based alloys. Moreover, the remarkably enhanced ductility of the RS + PHT processed A356 alloy permits further strengthening the material by work hardening as well as forming the material *via*. conventional solid-state forming techniques, which is expected to extend the applications of such an alloy.

## Results

### Simultaneous increase in strength and ductility of A356 alloy

Figure 2a shows the typically measured engineering stress-strain curves of A356 alloys subjected to RS at different cooling rates plus PHT treatment, where the strength and ductility of the RS + PHT treated A356 alloys increase simultaneously with increasing the cooling rate upon RS. Further T6 heat treatment on the RS + PHT processed alloys (*cf*. Methods for details of the procedures) does not change the trend of simultaneous increase in the strength and ductility of the alloys with increasing the cooling rate upon RS, see Fig. 2b,c. Mechanical properties of the alloys after different treatments are summarized in Table 1. It is shown that after the solid solution treatment, YS and UTS of the alloys increase slightly, whereas ETF is enhanced significantly; artificial aging on the solid solution treated alloys leads to pronounced increases in YS and UTS, as well as a certain reduction in ETF, see Table 1. The evolution of the mechanical properties of the alloys subjected to solid solution treatment and artificial aging shows similar behaviors with the casting A356 alloy processed normal route^16^. The corresponding mechanisms have been well elucidated in the literature^17^ and will therefore not be addressed here.

**Figure 1:**
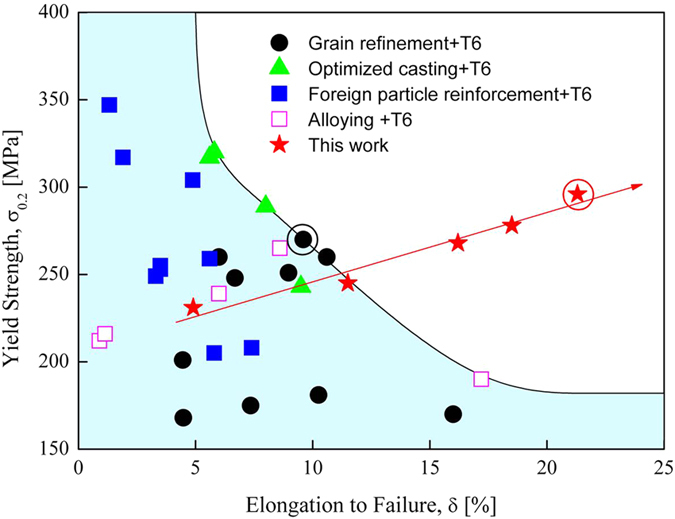
Yield strength (YS) and elongation to failure (ETF) of the A356 alloy achieved by various strengthening strategies: foreign particle reinforcement (blue closed squares^4^,^5^,^6^), grain refinement (black closed circles^7^,^8^), alloying (open squares^11^,^12^), and optimized casting (green closed triangles^11^,^12^). YS and ETF of A356 alloys obtained by combining the RS + PHT route with T6 heat treatment (red stars, the red arrow marks the direction of increasing cooling rate upon RS, the data point marked by the red circle represents the best combination of YS and ETF.). The black and red circles mark the best combination of YS and ETF obtained by rapid solidification at a cooling rate of 100 K/s and the subsequent T6 heat treatment^8^, and that achieved by combination of the current RS + PHT route with T6, respectively.

**Figure 2:**
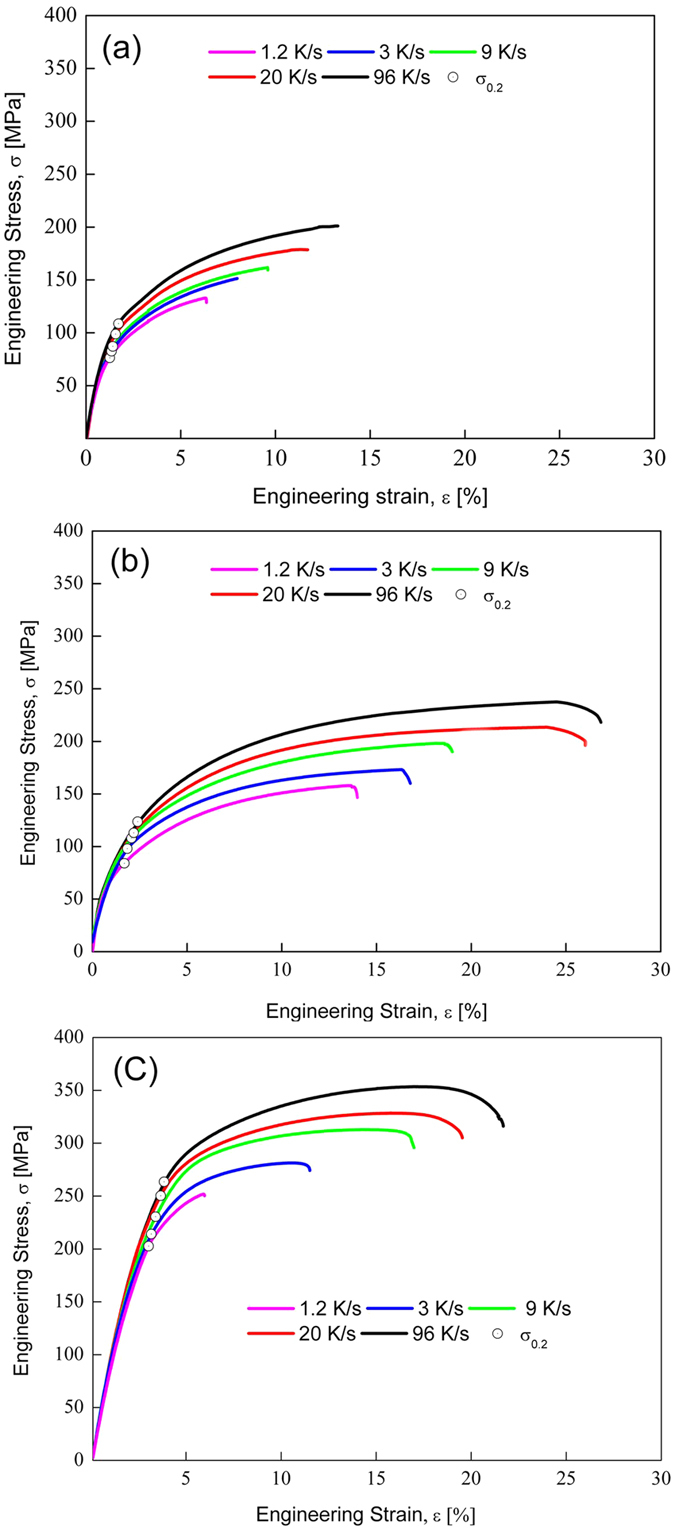
Measured engineering stress-strain curves of the A356 alloys processed by the RS+PHT and the subsequent T6 heat treatment. (**a**) RS + PHT treated, (**b**) solid solution treated at 813 K, and (**c**) artificially aged at 453 K. The curves shown in (**b**) and (**c**) correspond to the samples with peak YS values (*cf*. Supplementary Fig. S1). In order to show the changes in the mechanical properties in different treatment states, same scales of the coordinate axes are adopted in the three plots.

Figure 1 presents the comparisons of YS and ETF of the A356 alloy processed by application of RS + PHT route and T6 heat treatment with those achieved by other strategies combined also with T6 heat treatment. As the cooling rate is above 9 K/s, the combination of YS and ETF achieved by the current route goes beyond the region covering the data reported in the literatures^4^,^5^,^6^,^7^,^8^,^9^,^10^,^11^,^12^,^13^. With further increasing the cooling rate upon RS, the trend of increasing YS and ETF is enhanced systematically, showing exceedingly different characteristics compared to other strengthening approaches where the strength-ductility trade-off dilemma inevitably exists, as shown in Fig. 1. The results given above indicate clearly that the RS + PHT route developed in this work provides an efficient way in breaking through the strength-ductility trade-off dilemma for the A356 aluminum alloy.

### Hierarchical microstructure formed by RS + PHT

**Figure 3:**
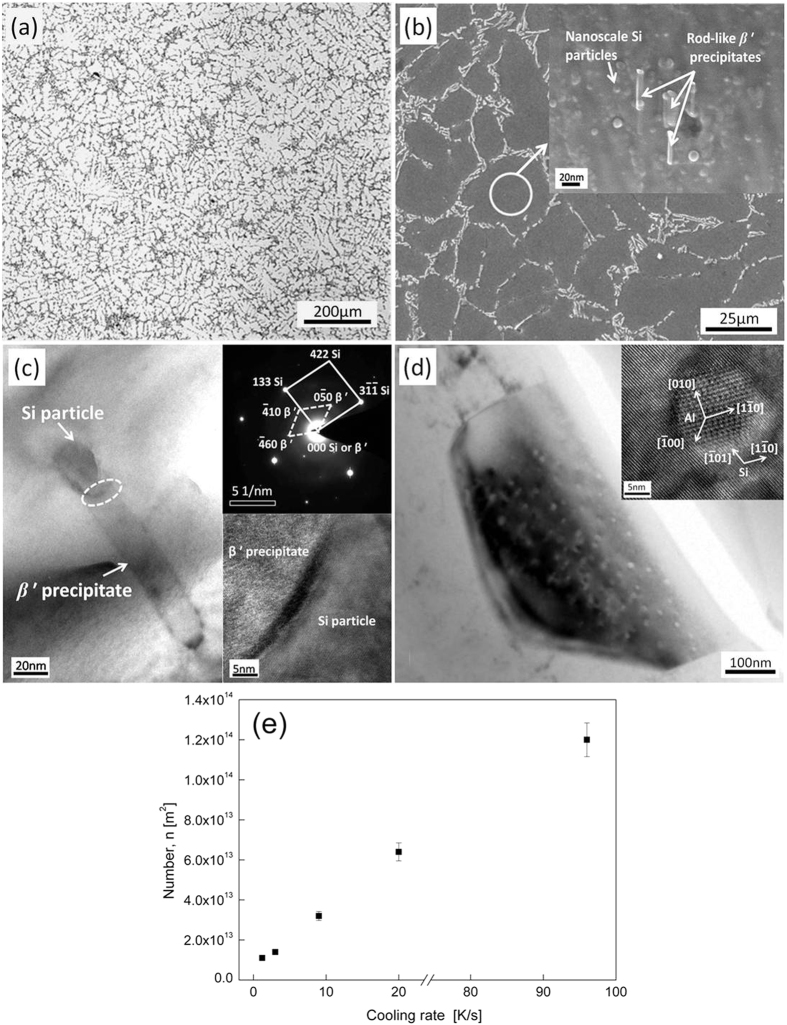
Microstructures of the RS and RS+PHT processed A356 alloys. (**a**) Typical morphology of the solidification microstructure of A356 alloys (cooling rate upon RS: 96 K/s). (**b**) SEM image of the A356 alloy processed by RS + PHT route (cooling rate upon RS: 96 K/s); the inset shows the highly dispersed nanoscale Si particles; a few Si particles are associated with rod-like β′ (Mg_9_Si_5_) phase. (**c**) TEM bright field image of a nanoscale Si particle associated with a β′ (Mg_9_Si_5_) phase; the upper and lower insets are the electron diffraction pattern taken from the selected area circled by the white dash line and the corresponding image at higher magnification; the cooling rate upon RS of the sample is 96 K/s. (**d**) TEM bright field image of the eutectic Si decorated by the nanoscale Al particles; the inset is the high resolution TEM image of an Al particle decorated in Si matrix; the cooling rate upon RS of the sample is 96 K/s. (**e**) The average density of nanoscale Si particles in the interior of Al dendrites measured by counting the number of particles in a specific area from at least three individual SEM images.

Solidification of A356 aluminum alloy consists of two stages: 1) primary solidification of Al dendrites at around 888 K, and 2) secondary solidification of eutectic phase (Al + Si) in inter-dendritic region at around 853 K, see Supplementary Fig. S2. A typical microstructure of as-solidified A356 alloys consists of coarse dendrites and eutectic structure in the inter-dendritic areas, see Fig. 3a. The coarse dendrites and the inter-dendritic phases are identified as Al phase and (Al + Si) eutectic phase, respectively, *cf*. Supplementary Fig. S3a,b. With increasing the cooling rate upon RS, the Al dendritic and (Al + Si) eutectic structures are subjected to continuous refinement, see Supplementary Fig. S4a–c.

The as-solidified microstructures of A356 alloys described above show similar characteristics to those processed by normal casting techniques. However, highly dispersed nanoscale Si particles with typical diameter of around 20 nm which are usually observed in the over-aged A356 alloy^18^ appear in Al dendrites (Fig. 3b,c), after PHT treatment at 473 K for 15 min. The density of these nanoscale Si particles is found to increase with increasing the cooling rate upon RS, see Fig. 3e. In samples solidified at a cooling rate above 9 K/s, part of these Si particles is associated with a rod-like *β*′ (Mg_9_Si_5_) phase, see Fig. 3c. Besides the Al dendrites, changes also appear in the eutectic Si in the samples solidified at high cooling rates (i.e. 20 K/s and 96 K/s), i.e. nanoscale particles with typical diameter of around 15 nm identified as Al phase are observed in eutectic Si phase, see Fig. 3d. The HRTEM image (inset in Fig. 3d) shows that the crystallographic orientation between the nanoscale Al particles and the eutectic Si matrix follows (110)_Al_//(110)_Si_ and [001]_Al_//[111]_Si_ which is in accordance with a previous report in ref. 19.

The formation of nanoscale Si and Al particles in A356 aluminum alloys has, although, been report in several previous literatures^18^,^19^,^20^, the effect of these structures has not been aroused enough attentions, because they are observed in either the overaged alloys^18^ or the alloys subjected to some complicated heat treatment procedures^19^,^20^. By simply inserting a PHT procedure between non-equilibrium solidification and conventional T6 heat treatment and adjusting the solidification rate *via*. alternating cooling rate upon RS, the formation of nanoscale Si and Al particles in A356 aluminum alloys can be well favored and will be shown to play crucial roles in breaking through the strength-ductility dilemma of the A356 alloys.

It is well known that rapid solidification exerts solute drag effect^21^. Therefore, the rapid solidification achieved by increasing the cooling rate will certainly lead to the trapping of Si element in Al and of Al element in Si upon solidification and make the matrix phases being supersaturated by these elements. Since the solubility of both Si element in Al dendrites and Al element in eutectic Si phase are only in ppm level at room temperature^22^, supersaturating them in the matrix phases is expected to provide large driving forces for their precipitation. Since the precipitation of either Si element from Al dendrites or Al element from eutectic Si phase is controlled by long range solute diffusion, these elements will then remain supersaturated in the matrix phases. The PHT treatment on the RS alloys at the moderate temperature favors the kinetics of precipitation, and therefore enables the formation of nanoscale Si particles in Al dendrites and nanoscale Al particles in eutectic Si phase. On the other hand, it has been reported that diffusion of solute can be mediated by non-equilibrium vacancies introduced by, e.g. quenching^23^,^24^. Regarding the high cooling rate achieved by cooling samples in Cu mould (*cf*. methods section), quenched-in non-equilibrium vacancies are expected to form, which may facilitate diffusion and clustering of the supersaturated Si and Al elements and favor the formation of these nanoscale particles in a short time period (15 min) at a relatively low temperature (473 K), refer to methods section for details. Furthermore, these vacancies will be expected to annihilate in sinks, once the nanoscale particles/precipitates form. When this occurs, further coarsening/dissolution of these particles upon heating will be suppressed, because the diffusion without assisted by vacancies is rather sluggish^23^. This may provide a possible explanation on the remarkable thermal stability of these nanoscale particles mentioned below. The increase in solidification rate by increasing the cooling rate upon RS will strengthen the solute trapping of Si element in Al dendrites and that of Al element in eutectic Si phase, which will certainly enhance the level of super-saturation. This can be reflected by the increased density of nanoscale Si particles in Al dendrites with increasing the cooling rate and the presence of nanoscale Al particles in eutectic Si phase at sufficiently high cooling rates (e.g. 20 K/s and 96 K/s).

**Figure 4:**
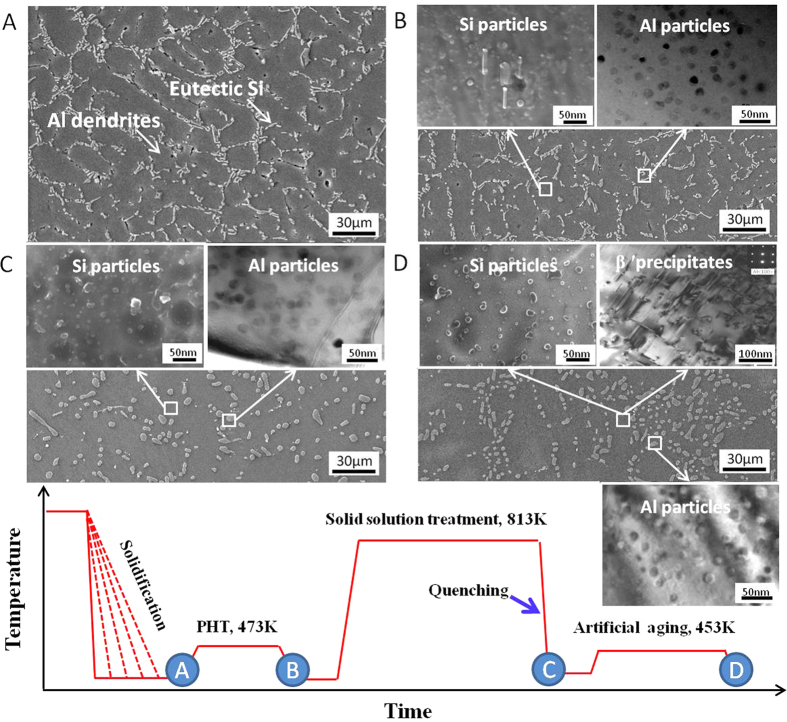
Development of microstructure of the A356 alloy solidified at a cooling rate of 96 K/s (shown in the upper part) processed by RS+PHT and the subsequent T6 heat treatment (shown in the lower part). The as-solidified microstructure consists mainly of Al dendrites and eutectic Si phase (**A**). After PHT treatment at 473 K, highly dispersed nanoscale Si particles and nanoscale Al particles appear in the Al dendrites and the eutectic Si phase, respectively (**B**). Further solid-solution treatment at 813K leads to the extensive spheroidization of eutectic Si, whereas, does not cause significant changes in these nanoscale particles (**C**). The artificial aging at 453K causes the precipitation of β′ phase in Al dendrites, and again, does not affect the presence of these particles (**D**).

Further applying T6 heat treatment on the RS + PHT processed alloys, extensive changes of the microstructures, e.g. spheroidization of eutectic Si phase and precipitation of β′ phase, are observed (Fig. 4), whereas the nanoscale particles in either the Al dendrites or the eutectic Si phase show remarkable thermal stability and are retained well, see Fig. 4. The remarkable thermal stability of these particles is crucial, since, as illuminated in the following section, these particles are believed to be the responsible factors for the simultaneous increase in strength and ductility with increasing the cooling rate upon RS for both the RS + PHT and RS + PHT + T6 processed alloys.

### Mechanisms for simultaneous increase of strength and ductility

The grain refinement achieved by RS^7^,^9^ or addition of grain refiner^9^,^10^ is also expected to increase concomitantly the strength and ductility of A356 alloy, but its effect is rather limited, see Fig. 1. The best combination of YS and ETF obtained by this strategy, viz. combining the RS at a cooling rate of around 100 K/s with T6 heat treatment, is 260 MPa and 11% (marked by the black circle in Fig. 1), which are remarkably lower than that achieved by combining the RS + PHT route with the T6 heat treatment (marked by the red circle in Fig. 1). Besides the effects of RS, there must be other mechanisms arising from the PHT treatment being responsible for the enhanced mechanical properties of the A356 alloy studied in this work. The increase of YS with increasing the cooling rate upon RS can be ascribed to the refinement of microstructure, enhanced solid solution hardening and enhanced hardening of β′ precipitates which has been well discussed elsewhere^25^,^26^,^13^. Also, the Orowan strengthening by the highly dispersed Si particles is also expected to contribute to the enhanced YS. These mechanisms have been well understood and will not be necessary to be addressed in details here. In the following, we will focus mainly on the remarkably enhanced ductility of the material.

**Figure 5:**
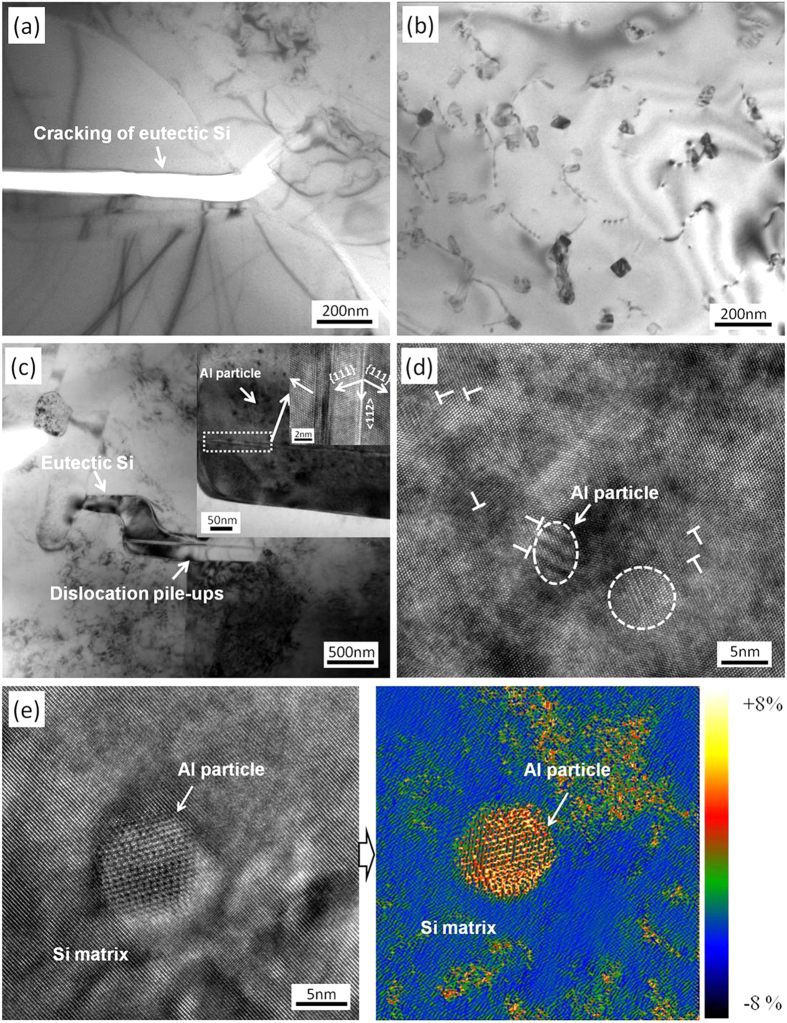
Microstructures of the samples subjected to tensile deformation. (**a**) Cracking of the eutectic Si formed in the sample solidified at 3 K/s after tensile deformation. (**b**) Interaction of dislocations and the Si particles in the tensile deformed sample, showing the pinning and storage of dislocations in the interior of Al matrix by Si particles. (**c**) The morphology of an eutectic Si in the tensile deformed sample; the magnified TEM bright field image and HRTEM image inserted in the upper right corner show the details of a deformation twin in the eutectic Si. (**d**) Dislocations in the vicinity of nanoscale Al particles decorated in eutectic Si. (**e**) A HRTEM image of the nanoscale Al particle decorated in Si matrix (left) and the corresponding strain map obtained by geometric phase analysis (right).

We first discuss the effect of nanoscale Si particles within the interior of Al dendrites. In A356 alloy, the plastic deformation ability of eutectic Si is usually rather limited, and plastic deformation of the alloy is dominated mainly by the dislocation activities in Al dendrites^3^. If the interior of Al dendrites is free or lack of dislocation obstacles, the dislocation pile-ups will occur easily in the Al dendrites/eutectic Si interface, causing the accumulation of local stress therein and leading to the cracking of the eutectic Si and eventually the fracture of the whole sample, see e.g. Fig. 5a. Under this condition, the alloy exhibits low ductility. This is exactly the case of the alloys solidified at low cooling rates, where the density of nanoscale Si particles is relatively low, even after the PHT treatment. With increasing the cooling rate, the density of Si particles in the interiors of Al dendrites increases continuously, see Fig. 3e. As shown in Fig. 5b, after plastic deformation, multiple dislocations are tangled by these Si particles in the interiors of Al dendrites, indicating that the particles enhance the dislocation storage in the interiors of Al dendrites. When this occurs, the plastic strain induced by deformation will be partitioned in the grain interiors rather than accumulated in the Al/Si interface. This will be essentially helpful in enhancing the deformation stability of the material and increase its ductility^27^,^28^,^29^. The effect of nanoscale Si particles on plastic deformation stability of ultrafine Al was also discussed by Huang *et al*.^30^,^31^, where it is shown that the presence of these particles enhances the deformation stability of ultrafine Al. This mechanism is believed to work as well for the nanoscale Si particles in the interior of Al dendrites. The Supplementary Fig. S5 shows the true stress-strain curves of the solid solution treated A356 alloys solidified at different cooling rates and the corresponding work hardening rates. It is seen that a higher cooling rate upon RS corresponds a higher work hardening rate. If the nanoscale Si particles are able to cause the storage of dislocations in the interiors of Al dendrites, the increased number of these particles by increasing cooling rate upon RS will certainly lead to the rise of work hardening rate.

The storage of dislocations by the nanoscale Si particles is also supported by our *in situ* TEM observations. Two typically living processes of interaction between dislocations and Si particles are recorded in Supplementary Movies S1 and S2. Movie S1 shows clearly that, upon plastic deformation, the dislocations are tangled and stored by the Si particles, which just confirms the above mechanism conferred by TEM observation of the tensile deformed sample (Fig. 5b). Movie S2 presents the dislocation activities in a region subjected to more intensive deformation. In the area free of Si particles, the dislocations slip quickly through the Al matrix; whereas in the area decorated with Si particles, the plastic deformation is more extensive and the dislocation activities therein are very active, suggesting that these particles can enhance strongly the deformation stability without significantly restricting the dislocation activities.

Besides the nanoscale Si particles, the changes of eutectic Si phase with the RS + PHT treatment are also expected to play important roles in delaying failure of the material. It is well known that eutectic Si phase is brittle, showing rather low tolerance to stress localization. Upon plastic deformation, it is very suspicious to cracking once stress localization occurs in the Al dendrites/eutectic Si interface. For the eutectic Si blocks with large sizes (e.g. the samples corresponding to the low cooling rates, *cf*. Supplementary Fig. S4a), the stress accumulation in the Al dendrites/eutectic Si interface will occur easily, because the stress development upon deformation cannot be distributed easily by other blocks. In contrast, the stress localization can be alleviated by the refinement of the eutectic Si blocks^32^,^33^. As shown in Supplementary Fig. S4, the increase of solidification rate has led to a significant refinement of the eutectic Si blocks. This will certainly be helpful for delaying the cracking and contribute partly to the enhanced ductility of the material. Besides this, what is more interesting is that the eutectic Si decorated with the nanoscale Al particles formed at the high cooling rates (i.e. 20 K/s and 96 K/s) is found to be deformable. Figure 5c shows a typical morphology of a eutectic Si decorated with nanoscale Al particles in a sample after tensile deformation, where dislocation pile-ups of Al dendrites are observed on the lower edge of the eutectic Si plate as marked by the white arrow. This indicates a high level of stress accumulated therein upon deformation. Along with the presence of these dislocation pile-ups, twinning of eutectic Si which is not observed in the un-deformed samples appears, see insert of Fig. 5c, suggesting twinning probably plays a role in the deformation of eutectic Si. On the other hand, it is shown by the high resolution TEM (Fig. 5d) that dislocations form around the nanoscale Al particles, which is considered as the other evidence for the plastic deformation of eutectic Si. This is also supported by the geometric phase analyses (GPA) proposed by Martin *et al*.^34^ on high resolution TEM images of the eutectic Si decorated with nanoscale Al particles (Fig. 5e), where an intensive strain contrast can be seen in the eutectic Si decorated with Al particles. This again indicates that the eutectic Si should be subjected to plastic deformation. Although the relevance between the ductilization of eutectic Si and the embedded nanoscale Al particles is not clear, the formation of twins and dislocations in the eutectic Si is apparently helpful in releasing the accumulated stress in the Al dendrites/eutectic Si interface and reducing the cracking tendency of the eutectic Si. This will contribute as well to the enhanced ductility of material significantly compared to the un-deformable Si.

## Discussion

Besides breaking through the strength-ductility trade-off dilemma of A356 alloy, the currently designed RS + PHT route also bring advantages in not only engineering microstructures of other Al-Si-based alloys but also extending the applications of A356 alloy.

Since the formation of the hierarchical microstructure being responsible for increasing simultaneously the strength and ductility depends only on the RS + PHT route, the current strategy of engineering microstructure may offer a general pathway in tailoring mechanical properties of Al-Si-based alloys.

Compared to the conventional process procedure of A356 alloy, i.e. solidification plus T6 heat treatment, the current route requires only increasing the cooling rate upon solidification and inserting one simple procedure, i.e. PHT, between solidification and T6 heat treatment. The trend of simultaneously enhancing the strength and ductility can be strengthened systematically by alternating only one parameter, i.e. cooling rate upon RS. It then follows that the simplicity in processing enables the possibility of applications for industrial scaling production.

The RS + PHT route enhances remarkably the ductility of A356 alloy. Inspired by this, further strengthening the material by applying multiple cold rolling plus T6 heat treatment was carried out by the authors. The preliminary results show that the mechanical property of A356 alloy can be improved significantly. As shown in Supplementary Fig. S6a, YS, UTS, and ETF of the as-treated A356 alloy reach 320 MPa, 500 MPa, and 11.3%, respectively, which approach or exceed those of many age hardened wrought aluminum alloys. The Supplementary Fig. S6b shows that the excellent ductility of the RS + PHT processed A356 alloy has substantially enhanced the workability of the alloy, thus enabling possible forming of the material by conventional solid-state compression, rolling and extrusion. If non-equilibrium solidification, deformation process, and heat treatment can be combined properly and optimized to establish a systematic processing route as suggested by one of the authors in a review article^35^, there will be still room to improve further the comprehensive mechanical properties and workability of A356 alloy. If so, the applications of A356 will be extended possibly to part of the fields where wrought aluminum alloys are used generally. This will be a direction of our future work. Also, considering the excellent castability and fluidity of A356 alloy melt compared to the common wrought aluminum alloys, the production of A356 may avoid involving the unnecessary procedures for eliminating solidification defects produced upon melting and casting, such as multiple hot rolling/forging being essential for production of common wrought alloys. This will certainly be helpful in reducing the cost of industrial production.

In summary, we have obtained hierarchical microstructure by designing a simple RS + PHT route to break successfully through the strength-ductility trade-off dilemma in an A356 aluminum casting alloy. By applying the RS + PHT route, YS and ETF of the A356 alloy increase simultaneously with increasing the cooling rate upon RS, which is ascribed to the hierarchical microstructure, i.e. nanoscale Si particles dispersed in the interiors of Al dendrites and nanoscale Al particles decorated in eutectic Si phase, formed upon RS + PHT treatment. The former enhances the work hardening of Al dendrites, while the latter causes the ductilization of eutectic Si. The hierarchical microstructure shows remarkable thermal stability against heating. This allows further improvement of the comprehensive mechanical properties of the RS + PHT processed A356 alloy *via*. T6 heat treatment without changing the trend of simultaneous increase in strength and ductility with increasing the solidification rate. The currently designed RS + PHT route brings significant advantages. First, the simplicity of the RS + PHT route makes it being suitable for industrial scaling production. Second, the strategy of engineering microstructure offers a general pathway in tailoring mechanical properties of other Al-Si-based alloys. Third, the excellent ductility of the RS + PHT processed alloy provides the opportunity for extending the applications of A356 alloy.

## Methods

### Materials processing

The materials used in this work is an Al-Si-based commercial A356 alloy with a nominal composition of Al-7.0Si-0.4Mg-0.1Fe in wt.%, where Mg is added intentionally to form the β′ (Mg_9_Si_5_) precipitates upon artificial aging which acts as a major strengthening precipitates in A356 alloy; while Fe is impurity which cannot be removed completely and will bringdetrimental effects on mechanical property of the alloy. 1.5 kg of alloy was melted in an electrical resistance furnace and degassed by hexachloroethane at 993 K under the protection of argon gas. After two cycles of degassing, the alloy melt was casted into a step-like Cu mould at 953 K to realize the rapid solidification with different cooling rates. Cooling curves upon solidification of the alloys were recorded by five individual K-type thermocouples pre-inserted in the Cu mould at the corresponding positions. The cooling rates of the alloy melts were determined from the recorded cooling curves. PHT treatment on the as-solidified alloys was carried out in a muffle furnace at 473 K for 15 min. The adopted temperature and time for PHT treatment were determined by balancing the working efficiency and the desired microstructures, i.e. ensuring the formation of highly dispersed fine particles in the matrix phases without changing significantly the as-solidified microstructures. T6 heat treatment was performed following the standard procedures for A356 alloy in the muffle furnace, i.e. solid solution treatment at 813 K and artificial aging at 453 K^36^. After solid solution treatment, the samples are subjected to water quenching. All heat treatments were carried out in the air. The peak solid solution treatment and artificial aging times were determined according to Supplementary Fig. S1a,b.

### Characterizations

Microstructures of the alloys were characterized by means of PMG3 Olympus optical microscopy, JSM-6460 scanning electron microscopy (SEM), and TecnaiG2 F30 transmission electron microscopy (TEM) operated at 300 kV. The compositional analyses were performed in an energy dispersive spectroscopy (EDS) equipped in the SEM. *In situ* TEM experiments were conducted in a Gatan 654 straining holder equipped in a JEM-2100 TEM operated at 200 kV. Upon *in situ* TEM investigations, the specimens were strained by controlling the total elongation *via*. a step motor in the straining holder. TEM and *in situ* TEM specimens were prepared by the electropolishing method in a double-jet apparatus in a solution of 30% nitric acid and 70% methanol at −30 °C.

### Tensile tests

Tensile tests were carried out in a Shimadzu AG-X (5 kN) universal tensile test machine with an initial strain rate of 1.67 × 10^−3^ s^−1^. The tensile specimens have a gauge length of 20 mm, and a width of 5 mm following the ASTME-8 standard. To ensure the reproducibility, the samples corresponding to each processing conditions were tested at least five times.

## Additional Information

**How to cite this article**: Dang, B. *et al*. Breaking through the strength-ductility trade-off dilemma in an Al-Si-based casting alloy. *Sci. Rep*. **6**, 30874; doi: 10.1038/srep30874 (2016).

## Supplementary Material

Supplementary Information

Supplementary Movie S1

Supplementary Movie S2

## Figures and Tables

**Table 1:** Mechanical properties of the A356 alloys subjected to RS + PHT and T6 heat treatments.

States	Properties	Cooling rate upon RS				
		1.2 K/s	3 K/s	9 K/s	20 K/s	96 K/s
RS + PHT	YS, MPa	75 ± 5	82 ± 5	87 ± 5	100 ± 5	110 ± 5
	UTS, MPa	133 ± 5	150 ± 5	165 ± 5	182 ± 5	202 ± 5
	ETF, %	6.3 ± 0.2	8 ± 0.2	9.5 ± 0.2	11.6 ± 0.2	13.2 ± 0.2
Solid solution treatment	YS, MPa	85 ± 5	98 ± 5	108 ± 5	113 ± 5	120 ± 5
	UTS, MPa	162 ± 5	173 ± 5	195 ± 5	214 ± 5	230 ± 5
	ETF, %	13.2 ± 0.2	16.3 ± 0.2	18.4 ± 0.2	26.5 ± 0.2	28.3 ± 0.2
Artificial aging	YS, MPa	231 ± 5	245 ± 5	268 ± 5	278 ± 5	296 ± 5
	UTS, MPa	251 ± 5	280 ± 5	311 ± 5	326 ± 5	353 ± 5
	ETF, %	4.9 ± 0.2	11.5 ± 0.2	16.2 ± 0.2	18.5 ± ± 0.2	21.3 ± 0.2
