# Supplementary material for: Breaking through the strength-ductility trade-off dilemma in an Al-Si-based casting alloy
Source: Sci Rep. 2016 Aug 9;6:30874. doi: 10.1038/srep30874 (PMC4977527; doi:10.1038/srep30874)
Supplement: Supplementary Information [file srep30874-s1.pdf]

Supplementary Materials

Breaking through the strength-ductility tradeoff dilemma in an Al-Si-based casting alloy

B. Dang[1], X. Zhang[1], Y.Z. Chen[1*], C.X. Chen[2], H.T. Wang[2], F. Liu[1*]

[1] State Key Laboratory of Solidification Processing, Northwestern Polytechnical University, 710072 Xi'an, P.R. China

[2] Institute of Applied Mechanics, Zhejiang University, 310027 Hangzhou, P.R. China

Supplementary Materials include:

Supplementary Figures S1-S6

Supplementary movie legends S1-S2

[*] Corresponding authors. email addresses: liufeng@nwpu.edu.cn (F. Liu), yzchen@nwpu.edu.cn (Y.Z. Chen), Tel: +86 29 88460374, Fax: +86 29 88491484

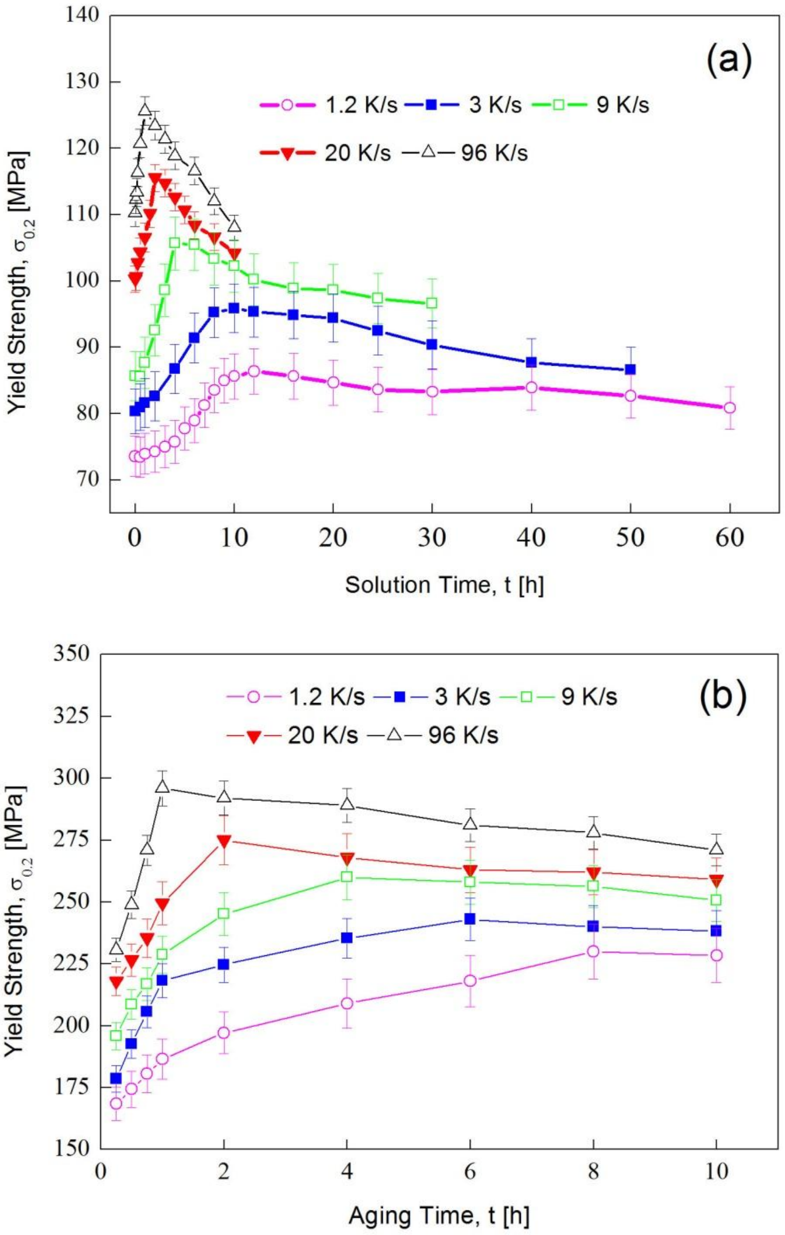

Figure S1. Evolution of YS of the RS+PHT processed A356 alloys subjected to solid solution treatment at 813 K (a), and artificial aging at 453 K (b). It is shown that the efficiency of T6 heat treatment is enhanced by increasing the cooling rate upon RS.

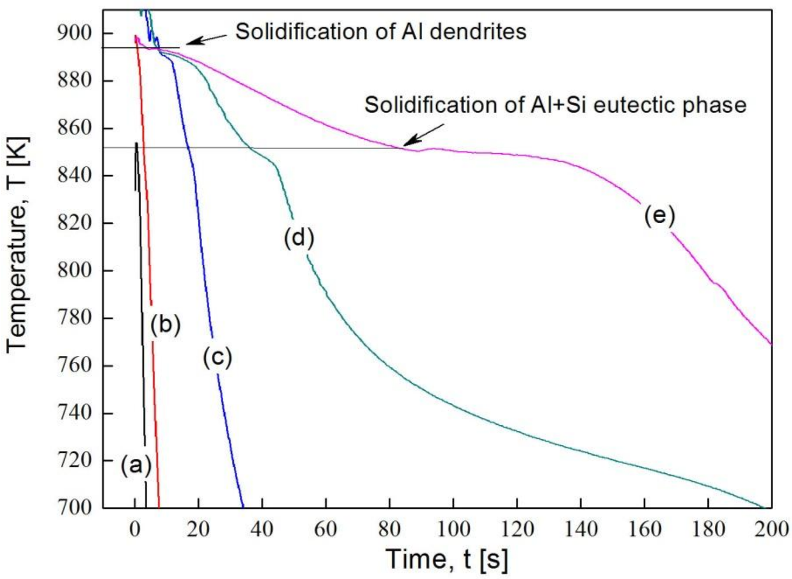

Figure S2. Cooling curves of A356 alloys subjected to solidification at different cooling rates measured by five individual thermocouples, (a) 96 K/s, (b) 20 K/s, (c) 9 K/s, (d) 3 K/s, and (e) 1.2 K/s. The inflexions appearing at 888 K and 853 K correspond to the recalescence events arising from the primary solidification of Al dendrites and secondary solidification of (Al+Si) eutectic phase, respectively. Limited by the responding time of the thermocouples, these inflexions are hardly observed in the cases of high cooling rates, i.e. 96 K/s and 20 K/s.

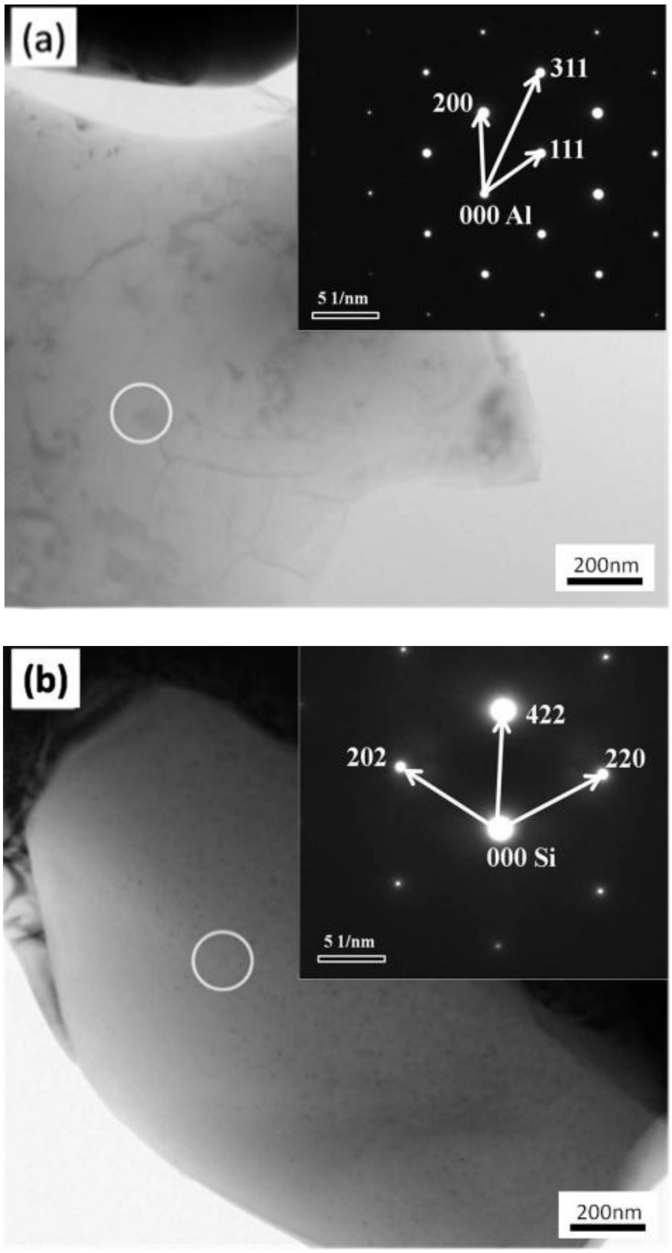

Figure S3 Typical TEM bright field image and the corresponding selection area electron diffraction pattern of Al dendrites (a) and eutectic Si (b).

Figure S4. Typical microstructures of the A356 alloys solidified at different cooling rates, (a) 1.2 K/s, (b) 9 K/s, and (c) 96 K/s.

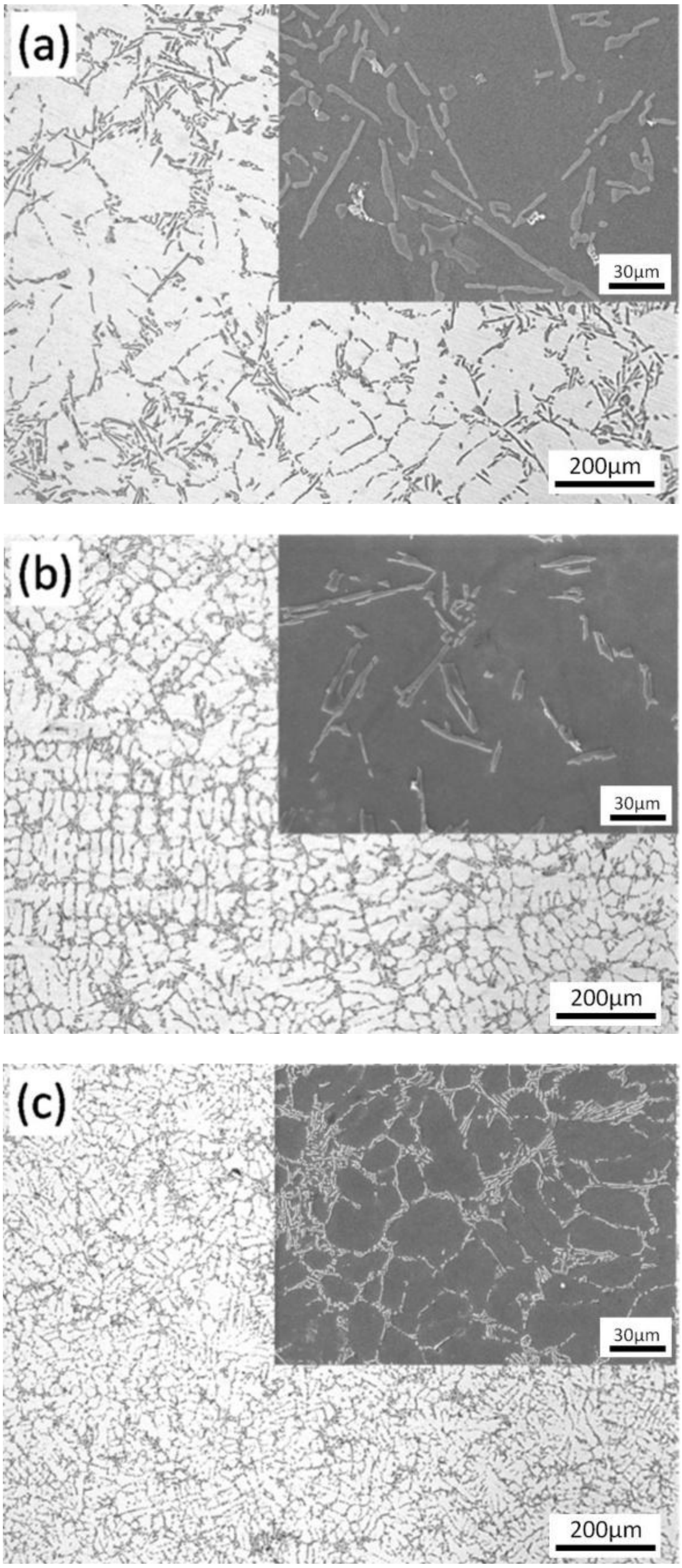

Figure S4. Typical microstructures of the A356 alloys solidified at different cooling rates, (a) 1.2 K/s, (b) 9 K/s, and (c) 96 K/s.

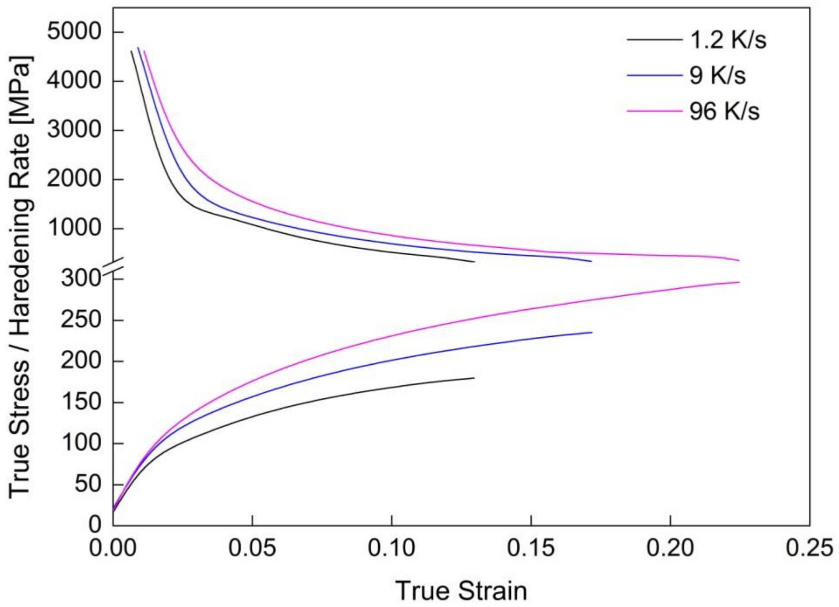

Figure S5. Typical true stress–strain curves converted from the engineering stress-strain curves and the corresponding to work hardening rate curves of the solid solution treated A356 alloys with respect to different cooling rates up RS. It is shown that with increasing the cooling rate upon RS, working hardening of the alloys is enhanced.

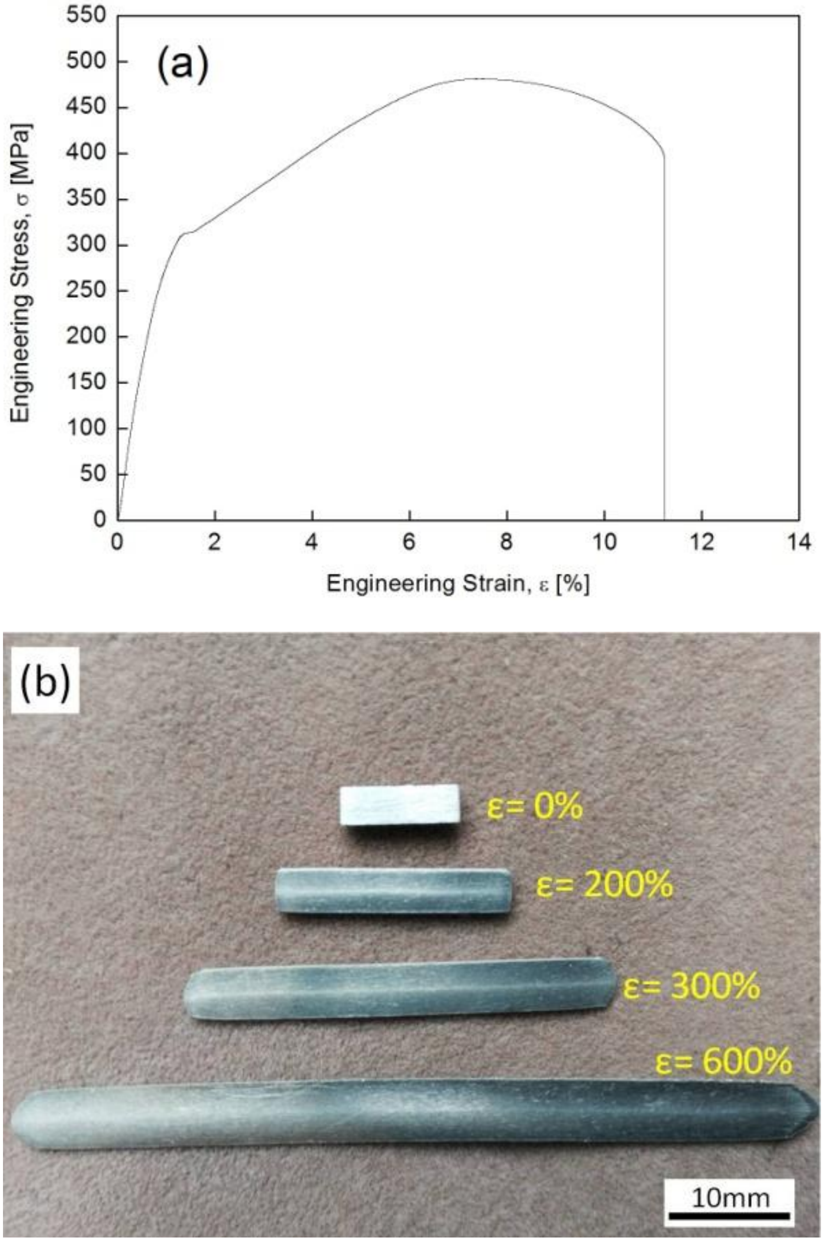

Figure S6. (a) Measured engineering stress–strain curves of the RS+PHT processed A356 alloy subjected to multiple cold rolling and subsequent T6 heat treatment. (b) Photographs of the sample subjected to multiple cold rolling show that the RS+PHT processed A356 alloy exhibits a remarkable workability.

Movie legends：

Movie S1. Interaction of dislocations of Al dendrites with the nanoscale Si particles, showing that upon deformation the dislocation is blocked and pinned by the Si particles. The white arrows point at three nanoscale Si particles.

Movie S2. Dislocation activities in a region of Al dendrites subjected to intensive plastic deformation, showing that in an area free of nanoscale Si particles (circled by the white solid line), the dislocations slip easily through the Al matrix; in an area decorated by Si particles (circled by the white dash line), the storage of dislocations by these particles occur, whereas the dislocation activities are still quite active.
